# Supplementary material for: New whaitsioids (Therapsida: Therocephalia) from the Teekloof Formation of South Africa and therocephalian diversity during the end-Guadalupian extinction
Source: PeerJ. 2017 Oct 5;5:e3868. doi: 10.7717/peerj.3868 (PMC5632541; doi:10.7717/peerj.3868)
Supplement: Supplemental Information 1 [file peerj-05-3868-s001.pdf]

ONLINE SUPPLEMENTARY INFORMATION

New whaitsioids (Therapsida: Therocephalia) from the Teekloof Formation of South Africa and therocephalian diversity during the end-Guadalupian extinction

ADAM K. HUTTENLOCKER[1,*] and ROGER M. H. SMITH[2,3]

[1] Department of Integrative Anatomical Sciences, University of Southern California, Los Angeles, California, U.S.A.;

[2]Evolutionary Studies Institute, University of the Witwatersrand, Johannesburg, South Africa;

[3]Iziko South African Museum, Cape Town, South Africa

*Corresponding author: *ahuttenlocker@gmail.com*

APPENDIX S1. List of 136 craniodental and postcranial characters scored for phylogenetic analysis of 56 therapsid taxa (including 49 therocephalians with inclusion of the new taxa) based on the most recent matrix of Huttenlocker et al. (2015) and Huttenlocker and Sidor (2016). Character 136 was newly added by Huttenlocker and Sidor (2016).

(1) Premaxilla: does not overhang incisor region (0); anterodorsal most tip of premaxilla forms a rostral process, overhanging incisors (1).

(2) Septomaxilla shape: long, narrow and moderately well exposed on facial region (0); enlarged and well exposed outside of external naris, broadly overlapping premaxilla anteriorly (1). (From Botha et al., 2007)

(3) External nare shape: moderately large and face anterolaterally (0); enlarged, close-set, and face more anteriorly (1).

(4) Dorsal profile of antorbital region: convex (0); relatively straight (1). (From Sidor and Hopson, 1998)

(5) Constriction of snout directly behind caniniform(s): absent (0); present (1). (From van den Heever, 1994)

(6) Preorbital depression: absent (0); present anterior to a thickened ridge on anterior margin of orbit, extending from lacrimal to canine buttress of maxilla (1).

(7) Maxilla facial plate (or 'dorsal lamina') shape: high (0); low with a height less than 40% its length (1).

(8) Maxilla facial plate concave ventral step: present between anterior-most maxillary teeth and incisors (0); absent (1).

(9) Broad excavation or pit in the maxilla immediately posterior to the dominant canine: absent (0); present (1).

(10) Posterior region of maxillary facial plate folded inward onto palatal region, so that maxilla is well exposed in ventral view just anterior to orbit: absent (0); present (1).

(11) Suborbital bar depth: shallow (0); deep (1). (From van den Heever, 1994)

(12) Suborbital bar lateral expansion: absent (0); slightly expanded and extends from jugal onto posterior part of maxillary facial plate, contributing to a pronounced degree of orbital convergence (i.e., orbits facing forward and appearing more triangular than oval in dorsal view) (1).

(13) Long, low rostrum just anterior to orbital region: absent (0); present (1).

(14) Anterior border of orbit location: posterior half of skull (0); anterior half of skull (1); near transverse midline (2). (Modified from van den Heever, 1994)

(15) Jugal postorbital process: present (0); absent (1).

(16) Postorbital bar: moderately well-built (0); extremely slender (1); absent/unossified (2).

(17) Ventral edge of zygomatic arch shape (behind orbit): distinct posteroventral orientation (0); relatively straight / horizontal (1); curved, having a concave ventral margin throughout its length (2).

(18) V-shaped, posterior border of nasals: absent (0); present, pointing toward occiput (1). (Modified from van den Heever, 1994)

(19) Median frontonasal crest: absent (0); present (1).

(20) Postfrontal: present (0); absent (1). (From Hopson and Barghusen, 1986; Sidor, 2001; Botha et al., 2007)

(21) Parietal (= pineal) opening: present irrespective of ontogenetic stage (0); absent or extremely reduced (1). (Modified from Rubidge and van den Heever, 1997; Botha et al., 2007)

(22) Temporal fenestra size in adults: fenestra and orbit subequal in size (0); fenestra larger than orbit (1). (Modified from Rubidge and Kitching, 2003)

(23) Zygomatic arch shape: moderately deep (0); very slender (1). (From Hopson and Kitching, 2001)

(24) Participation of parietal in temporal fenestra dorsal border: absent (0); present (1). (From Hopson and Barghusen, 1986)

(25) Parietal expanded posteriorly on the midline behind the region of parietal foramen: absent (0); present (1). (From Hopson and Barghusen, 1986)

(26) Parietal crest length: moderately long, just over half temporal fenestra length (0); short, less than half temporal fenestra length (1).

(27) Posterodorsal inclination of the temporal region reaching its maximum height where parietal crest meets lambdoidal (= occipital) crest: present (0); absent (1).

(28) Intertemporal width in adults: wide (0); narrow with vertical lateral faces (1). (Modified from Kemp, 1972b; Hopson and Barghusen, 1986)

(29) Temporal fenestra anteroposterior enlargement: absent (0); present (1). (From Sidor and Hopson, 1998)

(30) Squamosal posteroventral process: absent (0); present (1). (From Rybczynski, 2000)

(31) Medially directed process of squamosal contacting prootic: absent (0); present, enclosing pterygoparoccipital foramen (1). (From Kemp, 1972b, 1982)

(32) Parietal crest: short in adults, located posteriorly (0); extends forward to include parietal foramen (1). (From Hopson and Kitching, 2001.) Note: due to the ontogenetic development of this feature, we have not included an "absent" character state, but several taxa are instead coded "?" (i.e., the crest may or may not develop later).

(33) Nasal-lacrimal contact: absent (0); present (1). (From Hopson and Kitching, 2001; Botha et al., 2007)

(34) Prefrontal-postorbital contact in adults: absent (0); present only on dorsomedial wall of orbit (1); present dorsally, excluding frontal from orbit margin (2). (From Hopson and Kitching, 2001; Botha et al., 2007)

(35) Level of greatest width of zygomatic arches: toward middle (0); at back of arch (1). (From Hopson and Kitching, 2001)

(36) Upturning of alveolar margin of premaxilla: moderate to pronounced (0); absent or horizontal (1). (Adapted from Rubidge and van den Heever, 1997)

(37) Interorbital width: approximately 20% or more of total skull length (0); less than 20% of total skull length (1).

(38) Jugal anterior extent: extends anteriorly beyond anterior margin of orbit (0); restricted to anterior margin of orbit (1).

(39) Palatal fenestra for lower caniniform: absent (0); present and confluent with internal naris (1); a separate palatal housing for the lower canine is created by an extension of premaxilla and maxilla from the primary palate (2); a fossa for the lower canine is present where maxilla and premaxilla meet on the ventral surface of the secondary palate (3). (Modified from Hopson and Barghusen, 1986; van den Heever, 1994)

(40) Maxilla palatal processes: absent (0); form a well developed crista choanalis with a ridge extending posteriorly onto the palatine (1); contact or nearly contact the ventrally extending vomer at a shallow angle with no sutural connection (2); contact vomer with a strong but short sutural connection (3); contact vomer at a strong angle creating a concave anteromedial surface on the crista choanalis, and bearing a moderately long sutural connection with the lateral margins of the vomer (4); meet at midline, sharing a sutural connection and obscuring anterior portion of vomer on palatal surface (5); small anteriorly located processes contact transverse processes of vomer (6). (Modified from Hopson and Barghusen, 1986; van den Heever, 1994; Botha et al., 2007)

(41) Vomer shape between choanae: slightly bulbous, narrowing toward its contact with premaxilla (0); expands anteriorly and is widest at its contact with premaxilla (1); bears specialized transverse processes at level of upper caniniform(s) (2). (From Hopson and Barghusen, 1986; van den Heever, 1994)

(42) Vomer anteriormost contact with premaxilla: short contact with vomerine process of premaxilla (0); broadly overlaps ventral surface of premaxilla vomerine process (1). (From Hopson and Barghusen, 1986)

(43) Vomer fusion: paired (0); fused anteriorly (1); completely fused (2). (From Rubidge and Kitching, 2003)

(44) Vomer ventromedian crest between palatines: absent (0); present (1). (Modified from Kemp, 1972b; van den Heever, 1994)

(45) Palatine teeth: present (0); absent (1). (Modified from Sidor and Hopson, 1998; Rubidge and Kitching, 2003)

(46) Large suborbital vacuities bound by palatine, pterygoid, and ectopterygoid: absent in adults (0); present throughout ontogeny (1). (Modified from Hopson and Barghusen, 1986)

(47) Pterygoid median tubercle/crest: absent (0); ventromedian tubercle/crest is present anterior to interpterygoid vacuity (1). (Modified from van den Heever, 1994; Botha et al., 2007)

(48) Teeth on transverse processes of pterygoids: present (0); absent (1). (From van den Heever, 1994)

(49) Teeth on pterygoid boss: present (0); absent (1). (From van den Heever, 1994)

(50) Pterygoid transverse flange expansion: moderate (0); reduced (1); having sharp, posteriorly projecting wings with slight posterolateral extension (2).

(51) Pterygoid transverse flange position: anterior to center of orbit (0); posterior to center of orbit (1).

(52) Palatal exposure of maxilla behind canine: less than 20% distance from canine to anterior palatine suture (0); greater than 20% distance from canine to anterior palatine suture (1). (modified from Hopson and Kitching, 2001)

(53) Formation of (rudimentary) secondary palate: occurs such that posterior portions of the maxillae and palatines approach at midline, but are slightly open anteriorly, thus creating an incipient incisive fissure or foramen (0); anterior portion is more closed than the posterior, leaving no indication of an incisive foramen or fissure in forms with a complete secondary palate (1). *Biarmosuchus*, *Titanophoneus*, Gorgonopsidae, Anomodontia, and *Lycosuchus* are coded '?' for this character because basal representatives lack any indication of a secondary palate or crista choanalis. (Based on Maier, 1999)

(54) Parasagittal ridges on pterygoid: absent (0); present, running from medial posterior flare of transverse flanges to basioccipital (1). (Modified from van den Heever, 1994 and Rubidge and Kitching, 2003)

(55) Vomer anterior vault: present (0); absent (1). (Modified from Sidor and Hopson, 1998)

(56) Epipterygoid-parietal contact: separate from parietal (0); contacts parietal (1). (From Modesto et al., 1999)

(57) Epipterygoid ascending process: appears as a thin rod (0); slightly expanded anteroposteriorly (1); greatly expanded, the greatest dorsal anteroposterior length being almost equal to dorsal height (2). (Modified from Kemp, 1972b; Hopson and Barghusen, 1986; Botha et al., 2007)

(58) Posterior apophysis of epipterygoid contacting or nearly contacting the prootic: absent (0); present, enclosing an aperture presumably for trigeminal nerve (1). (From Kemp, 1972a; Hopson and Barghusen, 1986)

(59) Laterally directed processes of the prootic: absent (0); present, participating in pterygoparoccipital foramen (1). (From Hopson and Barghusen, 1986.)

(60) Basal tuber: small (0); large, ca. 1/3 occipital breadth (1). (From van den Heever, 1994)

(61) Dorsal surface of paroccipital process: relatively smooth or straight (0); deeply hollowed in floor of posttemporal fenestra (1). (From Hopson and Barghusen, 1986)

(62) Paroccipital process of opisthotic orientation: strongly posteroventral (0); moderately posteroventral (1); transverse relative to horizontal (2). (From Rubidge and Kitching, 2003)

(63) Opisthotic dorsolateral process: absent (0); present and contacts tabular and/or squamosal, thus excluding supraoccipital from post-temporal fenestra (1). (Modified from Botha-Brink and Modesto, 2011)

(64) 'Mastoid process': absent or poorly developed (0); squamosal and paroccipital process of opisthotic form a distinct, posteriorly projecting 'mastoid process' (1). (Based on Crompton, 1955)

(65) Tabular-opisthotic contact: present (0); tabular is withdrawn from contact with opisthotic (1). (From Modesto et al., 1999)

(66) Tabular contribution to post-temporal fenestra: extends below fenestra (0); just barely contacts post-temporal fenestra (1). (Modified from Sidor and Hopson, 1998)

(67) Trigeminal nerve exit: located between prootic incisure and epipterygoid (0); via a foramen between prootic and epipterygoid (1). (From Hopson and Kitching, 2001)

(68) Epipterygoid-prootic overlap: absent (0); present (1). (From Hopson and Kitching, 2001)

(69) Epipterygoid-frontal contact: absent (0); present (1). (From Hopson and Kitching, 2001)

(70) Occipital condyle: single (0); double (1). (From Hopson and Kitching, 2001; Botha et al., 2007)

(71) Tabular dorsal contribution to lambdoidal crest: low and broad with little or no contribution (0); high, nearly contacting dorsal margin of interparietal with significant contribution to lambdoidal crest (1).

(72) Quadrate and quadratojugal size: relatively large (0); reduced in height (1). (From Hopson and Barghusen 1986)

(73) Quadrate and quadratojugal situated in a depression on the anterior face of the squamosal: absent (0); present (1). (From Hopson and Barghusen, 1986; Sidor and Hopson, 1998)

(74) Stapes dorsal process: present (0); reduced/absent (1).

(75) Posteroventral process on quadrate in posterior notch (quadrate recess) of squamosal: absent (0); present (1). (From Hopson and Kitching, 2001; Botha et al., 2007)

(76) Stapedial foramen: oriented posteroventrally (0); dorsoventrally (1); absent (2). (Modified from Hopson and Kitching, 2001)

(77) Overall dentary shape: deep/robust (0); short and banana-shaped (1); long, slender, and relatively straight with a smooth ventral edge (2). (Modified from Hopson and Barghusen, 1986; Sidor, 2003)

(78) Dentary anterior portion: deepens anteriorly (0); continuously tapers to a narrow anterior edge (1). (Modified from Sidor, 2003)

(79) Specialized boss on posteroventral portion of dentary: absent (0); present (1).

(80) Dentary symphyseal region: only moderately expanded mediolaterally with a low mentum angulation in ventral view (0); anteroposteriorly thickened with strong suture (1). (Based on Mendrez, 1974b)

(81) Dentary lateral surface near canine: relatively smooth (0); bears a marked constriction behind lower canine where upper canine rests in dorsal and ventral views (1). (Based on Mendrez, 1974)

(82) Dentary angle anterolateral to reflected lamina: shallow, smoothly rounded (0); moderate or sharp (1); pronounced, protruding with an angle $< 120^{\circ}$ (2).

(83) Coronoid process posterodorsal terminal margin: straight (0); rounded (1); comes to a sharp point (2). (Modified from Sidor, 2003)

(84) Coronoid process dorsal extent: terminates below middle of orbit (0); or in upper half of orbit (1). (From Sidor, 2003)

(85) Dentary height increased posteriorly and postdentary bones reduced so that dentary forms a free standing coronoid process: absent (0); present (1). (Modified from Sidor and Hopson, 1998; Rubidge and Kitching, 2003)

(86) Postdentary bones height relative to total dentary height: equal (0); between 1/2 and equal (1). (Modified from Sidor and Hopson, 1998)

(87) Ventral margins of angular and dentary: confluent (0); angular (= tympanic) positioned dorsal to ventral margin of dentary (1). (From Hopson and Barghusen, 1986; Sidor and Hopson, 1998)

(88) Splenial: exposed laterally near symphysis (0); obscured laterally by dentary (1). (From Sidor and Hopson, 1998; Sidor, 2003)

(89) Mandibular fenestra: absent (0); penetrating mandible and visible laterally (1); surangular above and prearticular below a small fenestra on medial surface of mandible (2). (Modified from Kemp, 1972a,b; Gauthier et al., 1988; Sidor and Hopson, 1998)

(90) Reflected lamina shape and ventral extent: rounded, projecting below ventral margin of dentary at about the level of the second groove (0); slightly anteroposteriorly elongate (spade-shaped) and does not appear to extend below dentary (1); reduced and spoon-shaped (2).

(91) Dentary masseteric fossa in adults: absent (0); present, high on coronoid process (1). (From Hopson and Kitching, 2001; Botha et al., 2007)

(92) Reflected lamina of angular (= tympanic) size: large (0); reduced (1). (Modified from Hopson and Kitching, 2001)

(93) Area between left and right dentaries: widens greatly posteriorly (0); remains relatively long and narrow (almost slit-like) just posterior to symphyseal region (1).

(94) Dentary ramus lateral groove: absent (0); present (1). (‘Longitudinal depression’ of Botha et al., 2007)

(95) Interlocking incisors: present (0); absent (1). (Modified from Rubidge and Kitching, 2003; Sidor and Welman, 2003)

(96) Upper incisors: few, up to five (0); six (1); seven or more (2).

(97) Upper incisor longitudinal facets or fluting: absent (0); present (1). (From Hopson and Barghusen, 1986)

(98) Incisor shape: relatively straight and conical (0); spatulate, concave lingually with mesiolingual and distolingual crests (1). (From Hopson and Barghusen, 1986)

(99) Functional upper precanine maxillary teeth in adults: absent (0); present (1). (Modified from Hopson and Barghusen, 1986; van den Heever, 1994; Botha et al., 2007)

(100) Upper dominant canine size: large relative to maxillary height (0); medium (1); reduced even in adults (2). (From Rubidge and van den Heever, 1997)

(101) Deep groove running vertically along the anterior surface of the dominant canine: absent (0); present (1).

(102) Upper postcanines in adults: numerous, more than five (0); few, five or less (1); absent (2). (Modified from Kemp, 1972b; van den Heever, 1994)

(103) Lower canine: large (0); medium (1); reduced, even in adults (2). (Modified from Botha et al., 2007)

(104) Postcanine teeth in lower jaw: present (0); absent (1).

(105) Incisor cutting margins: serrated (0); unserrated/smoothly ridged (1). (From Hopson and Kitching, 2001)

(106) Postcanine apex shape (if applicable): tall, sharp apices (0); bear two or more cusps in line (1); fat, lanceolate cones (2).

(107) Lower incisors number: four (0); less than four (1); more than four (2).

(108) Relative length of maxillary tooth row: > 50% of total maxillary length (0); approximately 50% or less (1).

(109) Caudal vertebrae: more than fifteen (0); fifteen or less (1).

(110) Scapular blade shape: short and broad (0); moderate breadth (1); delicate and narrow (2).

(111) Procoracoid foramen position: bound within procoracoid (0); between procoracoid-coracoid suture (1); between procoracoid-scapula suture (2).

(112) Ossified cleithrum: present (0); absent (1).

(113) Interclavicle shape: long and thin (0); short and broad anterior to sternum (1).

(114) Humerus shape: short and robust (0); long and slender (1).

(115) Ectepicondylar foramen of humerus: present (0); absent (1).

(116) Manual digit III second phalanx: short or disc-like but present (0); absent (1).

(117) Manual digit IV phalangeal number: 5 (0); 4 (1); 3 (2).

(118) Sharp anterior process of the dorsal iliac plate: absent (0); present below an anterodorsal concavity (1). (Based on Kemp, 2005)

(119) Pubis and ischium orientation: relatively vertical (0); more horizontal, forming a broad puboischiatic plate (1). (Based on Kemp, 2005)

(120) Obturator foramen size: small (0); moderately enlarged (1); extremely large opening (2). (From Sidor and Hopson, 1998)

(121) Obturator foramen position: situated completely within pubis (0); bound by pubis and ischium (1).

(122) Femur bears distinct trochanter minor: absent (0); present (1). (Based on Kemp, 2005)

(123) Clavicle shape medially: broad (0); narrow (1). (Gauthier et al., 1988)

(124) Humeral head: convex articular surface extends broadly across head (0); articular surface bulbous and inflected (1). (Gauthier et al., 1988)

(125) Radius width relative to ulna distally: equal, or radius narrower (0); radius broader (1). (Gauthier et al., 1988)

(126) Femoral head shape: elongate to subspherical (0); oblong and spherical (1). (Gauthier et al., 1988)

(127) Calcaneal tuber (tuber calcis): absent (0); present and oriented posteriorly (1). (Gauthier et al., 1988)

(128) Premaxillary foramina on palate: two small foramina (0); two enlarged foramina (1); medially positioned premaxillary fossa present (2).

(129) Angle of incidence between adjoining processes of maxilla and vomer on surface of secondary palate: shallow so that anterior portion of choana rapidly narrows (0); smoothly arching along anterior border of choana (1) in ventral view.

(130) Parietal crest fusion at midline: forms narrow sagittal crest joined tightly around or behind a bulbous pineal housing (0); weak such that parasagittal contributions of left and right parietals are continuous with temporal line and bound a narrow fissure (1).

(131) Maxillary postcanine alveolar margin in ventral view: straight to slightly concave along buccal margin, bowing inward toward the midline (0); buccally convex at anterior extent, bowing outward away from the midline (1).

(132) Upper postcanine diastema immediately behind dominant caniniform: present, greater than the spacing between successive postcanines (0); absent (1).

(133) Lacrimal pits/foramina exposed ventrolaterally outside orbital wall in separate antorbital fossa: absent (0); present (1).

(134) Suborbital vacuity shape: suboval, about as long as wide (0); elongated and D-shaped (or subtriangular) with straight medial margins (1); broad, anteroposteriorly shorter than wide due to anterior truncation by scalloped webbing (2).

(135) Premaxilla vomerine process contacts maxilla on medial border of lower canine fossa: absent (thereby permitting vomer to contact anterior choana/canine fossa) (0); present (restricting vomer from direct contact with anterior choana/canine fossa).

(136) Epipterygoid processus ascendens orientation in lateral view: tilted posterodorsally (0); tilted anterodorsally (1).

APPENDIX S2. Character scoring for *Microwhaitsia* and *Ophidostoma* (originally referred to as '*Tropidostoma* AZ whaitsiid' and '*Cistecephalus* AZ hofmeyriid' respectively) based on matrix of Huttenlocker and Sidor (2016). List of 136 craniodental and postcranial characters corresponds to Appendix S1. Corrections include: char. 10 changed to '-' in *Euchambersia* (as autapomorphic condition of maxillary pit renders this character not applicable, rather than plesiomorphic); char. 19 changed to '1' in *Microwhaitsia*; char. 34 changed to '0' in *Ophidostoma*; char. 40 changed to '1' in *Ophidostoma*; char. 43 changed to '0/1' in *Hofmeyria atavus* (which shows the primitive state in BP/1/1399); char. 49 changed to '0' in *Ophidostoma*; char. 89 changed to '1' in *Ophidostoma*; char. 90 changed to '1' in *Ophidostoma*; char. 92 changed to '0' in *Ophidostoma*; char. 97 changed to '1' in *Microwhaitsia*. Character 136 was newly added by Huttenlocker and Sidor (2016). The complete character matrix is provided as an online supplementary data file in Nexus format.

```
'Microwhaitsia'
1001000001110?002011????????????00?1?111101111?????01?????????????????
??????1100011111111?0?1010100001001000????????????????????-?001?-?

'Ophidostoma'
???1000?0101010120010111110111?0001?0111?????1?1021011?????0??????????
??????1?0?01111111110011???????000?0?0????????????????????-000000?
```

APPENDIX S3. PAUP output.

Strict consensus of 1160 trees:

```
/-------------------------------------------------------------------- Biarmosuchus_tener
|
|  /----------------------------------------------------------------- Titanophoneus_potens
|  |
|  |   /------------------------------------------------------------- Gorgonopsia
\--+   |
   |   |  /---------------------------------------------------------- Anomodontia
   \---+  |
       |  |                                                  /------- Charassognathus_gracilis
       |  |                                                  |
       |  |  /-----------------------------------------------+   /--- Dvinia_prima
       \--+  |                                               \---+
          |  |                                                   \--- Procynosuchus_delaharpeae
          |  |
          |  |   /--------------------------------------------------- Lycosuchus_vanderrieti
          |  |   |
          \--+   |                                           /------- Glanosuchus_macrops
             |   |                                           |
             |   |                                           +------- Ictidosaurus_angusticeps
             |   |                                           |
             |   |                                           +------- Pristerognathus_polyodon
             |   |  /----------------------------------------+
             \---+  |                                        +------- Scylacosaurus
                 |  |                                        |
                 |  |                                        |   /--- Alopecodon
                 |  |                                        \---+
                 |  |                                            \--- Pardosuchus
                 |  |
                 |  |  /--------------------------------------------- Scylacosuchus
                 |  |  |
                 \--+  |                                     /------- Perplexisaurus
                    |  |                                     |
                    |  |                     /---------------+   /--- Chthonosaurus
                    |  |                     |               \---+
                    |  |                     |                   \--- Ichibengops
                    |  |   /-----------------+
                    |  |   |                 |  /-------------------- Annatherapsidus
                    |  |   |                 |  |
                    |  |   |                 \--+  /----------------- Cist_AZ_akidnog_USNM_PAL_412421
                    \--+   |                    |  |
                       |   |                    \--+   /------------- Akidnognathus_parvus
                       |   |                       |   |
                       |   |                       |   |         /--- Promoschorhynchus
                       |   |                       \---+  /------+
                       |   |                           |  |      \--- Olivierosuchus_parringtoni
                       |   |                           \--+
                       |   |                              |  /------- Euchambersia_mirabilis
                       |   |                              |  |
                       |   |                              \--+   /--- Cerdosuchoides
                       |   |                                 \---+
                       \---+                                     \--- Moschorhinus_kitchingi
                           |
                           |                    /-------------------- Ophidostoma
                           |                    |
                           |                    |            /------- Hofmeyria_atavus
                           |  /-----------------+            |
                           |  |                 |  /---------+   /--- Ictidostoma_hemburyi
                           |  |                 |  |         \---+
                           |  |                 |  |             \--- Mirotenthes_digitipes
                           |  |                 \--+
                           |  |                    |   /------------- Ictidochampsa_platyceps
                           |  |                    |   |
                           |  |                    \---+  /---------- Microwhaitsia
                           |  |                        |  |
                           |  |                        \--+  /------- Viatkosuchus_sumini
                           |  |                           |  |
                           \--+                           \--+   /--- Moschowhaitsia_vjuschkovi
                              |                              \---+
                              |                                  \--- Theriognathus_microps
                              |
```

```
|   /--------------------------------- Ictidosuchus_primaevus
|   |
|   +--------------------------------- Ictidosuchoides_longiceps
|   |
|   +--------------------------------- Ictidosuchops_rubidgei
\---+
    |  /------------------------------ Regisaurus_jacobi
    |  |
    |  +------------------------------ Urumchia
    |  |
    \--+                          /--- Karenites_ornamentatus
       |  /-----------------------+
       |  |                       \--- Mupashi
       |  |
       \--+   /----------------------- Lycideops_longiceps
          |   |
          |   |  /-------------------- Choerosaurus_dejageri
          \---+  |
              |  |                /--- Tetracynodon_tenuis
              \--+  /-------------+
                 |  |             \--- Tetracynodon_darti
                 |  |
                 \--+   /------------- Scaloposaurus_constrictus
                    |   |
                    |   |         /--- Ericiolacerta_parva
                    \---+  /------+
                        |  |      \--- Silphedosuchus
                        |  |
                        |  |  /------- Nothogomphodon
                        \--+  |
                           |  |   /--- Ordosiodon
                           |  +---+
                           |  |   \--- Hazhenia
                           \--+
                              |   /--- Antecosuchus
                              |   |
                              |   +--- Bauria_cynops
                              \---+
                                  +--- Traversodontoides
                                  |
                                  \--- Microgomphodon_oligocynus
```

```
Statistics derived from consensus tree:

     Component information (consensus fork) = 45 (normalized = 0.833)
     Nelson-Platnick term information = 609
     Nelson-Platnick total information = 654
     Mickevich's consensus information = 0.362
     Colless weighted consensus fork (proportion max. information) = 0.425
     Schuh-Farris levels sum = 11116 (normalized = 0.401)
     Rohlf's CI(1) = 0.897
     Rohlf's -ln CI(2) = 191.605 (CI(2) = 6.12e-84)
```

APPENDIX S4. MrBayes output.

Clade credibility values:

Subtree rooted at node 91:

```
                          /-------------------------------- Ophidostoma (20)
                          |
                          |                          /----- Ictidostomahem~ (27)
                          |                    /--59-+
                          |                    |     \----- Mirotenthesdig~ (30)
     /---------89---------+     /------80------+
     |                    |     |              \----------- Hofmeyriaatavus (29)
     |                    |     |
     |                    |     |         /---------------- Microwhaitsia (28)
     |                    \--52-+         |
     |                          |    /-76-+    /----------- Viatkosuchussu~ (32)
     |                          |    |    |    |
     |                          |    |    \-91-+     /----- Moschowhaitsia~ (33)
     |                          \-56-+         \--89-+
     |                               |               \----- Theriognathusm~ (34)
     |                               |
--77-+                               \-------------------- Ictidochampsap~ (31)
     |
     |     /----------------------------------------------- Ictidosuchuspr~ (35)
     |     |
     |     |----------------------------------------------- Ictidosuchoide~ (36)
     |     |
     |     |----------------------------------------------- Ictidosuchopsr~ (38)
     |     |
     |     |                                         /----- Regisaurusjaco~ (39)
     |     |    /-----------------61-----------------+
     \--91-+    |                                    \----- Urumchia (40)
           |    |
           |    |                              /----------- Choerosaurusde~ (41)
           |    |                              |
           |    |                         /-64-+     /----- Tetracynodonte~ (45)
           |    |                         |    \--76-+
           |    |                    /-59-+          \----- Tetracynodonda~ (46)
           \-84-+                    |    |
                |                    |    \---------------- Lycideopslongi~ (42)
                |    /-------55------+
                |    |               |               /----- Karenitesornam~ (43)
                |    |               \-------85------+
                |    |                               \----- Mupashi (44)
                |    |
                \-59-+    /-------------------------------- Scaloposaurusc~ (47)
                     |    |
                     |    |                          /----- Ericiolacertap~ (48)
                     |    |     /---------96---------+
                     \-86-+     |                    \----- Silphedosuchus (49)
                          |     |
                          |     |         /---------------- Nothogomphodon (50)
                          |     |         |
                          \--85-+         |          /----- Antecosuchus (53)
                                |    /-54-+          |
                                |    |    |    /--55-+----- Traversodontoi~ (55)
                                |    |    |    |     |
                                |    |    \-93-+     \----- Microgomphodon~ (56)
                                \-91-+         |
                                     |         \----------- Bauriacynops (54)
                                     |
                                     |               /----- Ordosiodon (51)
                                     \-------53------+
                                                     \----- Hazhenia (52)
```

Subtree rooted at node 96:

```
                                               /----------- Charassognathus~ (5)
                                               |
     /-------------------100-------------------+     /----- Dviniaprima (6)
     |                                         \--94-+
     |                                               \----- Procynosuchusde~ (7)
     |
```

```
           |     /----------------------------------------------- Lycosuchusvande~ (8)
           |     |
           |     |----------------------------------------------- Alopecodon (9)
           |     |
      --100+     |                                         /----- Glanosuchusmac~ (10)
           |     |--------------------58-------------------+
           |     |                                         \----- Pristerognathu~ (13)
           |     |
           |     |----------------------------------------------- Ictidosaurusan~ (11)
           |     |
           |     |----------------------------------------------- Pardosuchus (12)
           \--68-+
                 |----------------------------------------------- Scylacosaurus (14)
                 |
                 |    /------------------------------------------ Scylacosuchus (15)
                 |    |
                 |    |         /-------------------------------- Annatherapsidus (16)
                 |    |         |
                 |    |         |     /-------------------------- CistAZakidnogU~ (17)
                 |    |    /-83-+     |
                 |    |    |    |     |    /--------------------- Akidnognathusp~ (18)
                 |    |    |    \--95-+    |
                 \-68-+    |          |    |         /----------- Euchambersiami~ (19)
                      |    |          |    |         |
                      |    |          \-99-+    /-98-+     /----- Cerdosuchoides (23)
                      |    |               |    |    \--77-+
                      |    |               |    |          \----- Moschorhinuski~ (24)
                      |    |               \-100+
                      |    |                    |          /----- Promoschorhync~ (21)
                      \-65-+                    \----60----+
                           |                               \----- Olivierosuchus~ (22)
                           |
                           |------------------------------------- (91)
                           |
                           |                               /----- Chthonosaurus (25)
                           |---------------93--------------+
                           |                               \----- Ichibengops (26)
                           |
                           \------------------------------------- Perplexisaurus (37)
```

Root part of tree:

```
      /---------------------------------------------------------- Biarmosuchusten~ (1)
      |
      |---------------------------------------------------------- Titanophoneuspo~ (2)
      +
      |                  /--------------------------------------- Gorgonopsia (3)
      |                  |
      \--------99--------+                   /------------------- Anomodontia (4)
                         \--------100--------+
                                             \------------------- (96)
```

Phylogram (based on average branch lengths):

```
/- Biarmosuchusten~ (1)
      |
      |-- Titanophoneuspo~ (2)
      |
      |  / Gorgonopsia (3)
      +  |
      |  |       /-- Anomodontia (4)
      |  |       |
      |  |       |                             / Charassognathus~ (5)
      \--+       |                             |
         |       |           /-----------------+ / Dviniaprima (6)
         |       |           |                 \-+
         |       |           |                   \ Procynosuchusde~ (7)
         |       |           |
         \-------+           |      /---- Lycosuchusvande~ (8)
                 |           |      |
                 |           |      |- Alopecodon (9)
                 |           |      |
                 |           |      |- Glanosuchusmac~ (10)
```

```
|           |      |
|           |      |- Pristerognathu~ (13)
\-----------+      |
            |      | Ictidosaurusan~ (11)
            |      |
            |      | Pardosuchus (12)
            |      |
            |      | Scylacosaurus (14)
            |      |
            |      |  /- Scylacosuchus (15)
            |      |  |
            |      |  |  / Annatherapsidus (16)
            |      |  |  |
            \------+  |  |/- CistAZakidnogU~ (17)
                   |  | /+|
                   |  | |||/- Akidnognathusp~ (18)
                   |  | |\+|
                   |  | | ||   /----- Euchambersiami~ (19)
                   |  | | ||   |
                   |  | | \+ /-+/ Cerdosuchoides (23)
                   |  | |  | | \+
                   |  | |  | |  \ Moschorhinuski~ (24)
                   |  | |  \-+
                   |  | |    |/ Promoschorhync~ (21)
                   |  | |    \+
                   |  | |     \ Olivierosuchus~ (22)
                   |  | |
                   |  | |    /- Ophidostoma (20)
                   |  | |    |
                   |  | |    | /- Ictidostomahem~ (27)
                   |  | |    | |
                   \--+ |    | |- Mirotenthesdig~ (30)
                      | |  /-+/+
                      | |  | ||\ Hofmeyriaatavus (29)
                      | |  | ||
                      | |  | || /-- Microwhaitsia (28)
                      | |  | \+ |
                      | |  |  |/+ /- Viatkosuchussu~ (32)
                      | |  |  ||| |
                      | |  |  ||\-+/ Moschowhaitsia~ (33)
                      | |  |  \+  \+
                      | |  |   |   \-- Theriognathusm~ (34)
                      | |  |   |
                      | |--+   \- Ictidochampsap~ (31)
                      | |  |
                      | |  |    / Ictidosuchuspr~ (35)
                      | |  |    |
                      | |  |    | Ictidosuchoide~ (36)
                      | |  |    |
                      | |  |    | Ictidosuchopsr~ (38)
                      | |  |    |
                      | |  |    |  /- Regisaurusjaco~ (39)
                      | |  |    | /+
                      \-+  \----+ |\ Urumchia (40)
                        |       | |
                        |       | |    /-- Choerosaurusde~ (41)
                        |       | |    |
                        |       | |   /+/ Tetracynodonte~ (45)
                        |       | |   |\+
                        |       | |  /+ \- Tetracynodonda~ (46)
                        |       \-+  ||
                        |         |  |\- Lycideopslongi~ (42)
                        |         |/-+
                        |         || |/- Karenitesornam~ (43)
                        |         || \+
                        |         ||  \ Mupashi (44)
                        |         ||
                        |         \+  / Scaloposaurusc~ (47)
                        |          |  |
                        |          |  |  / Ericiolacertap~ (48)
                        |          |  | /+
                        |          \--+ |\ Silphedosuchus (49)
                        |             | |
                        |             | |   /-- Nothogomphodon (50)
                        |             | |   |
                        |             \-+   | / Antecosuchus (53)
```

```
                                |               |  /+ |
                                |               |  ||/+- Traversodontoi~ (55)
                                |               |  ||||
                                |               |  |\+\ Microgomphodon~ (56)
                                |               \--+ |
                                |                  | \- Bauriacynops (54)
                                |                  |
                                |                  |/ Ordosiodon (51)
                                |                  \+
                                |                   \ Hazhenia (52)
                                |
                                |  /- Chthonosaurus (25)
                                |--+
                                |  \- Ichibengops (26)
                                |
                                \--- Perplexisaurus (37)

|-----------| 0.500 expected changes per site

Calculating tree probabilities...

Credible sets of trees (14985 trees sampled):
   50 % credible set contains 7484 trees
   90 % credible set contains 13485 trees
   95 % credible set contains 14235 trees
   99 % credible set contains 14835 trees
```

REFERENCES CITED IN SUPPLEMENT

Botha, J., F. Abdala, and R. Smith. 2007. The oldest cynodont: new clues on the origin and early diversification of Cynodontia. Zoological Journal of the Linnean Society 149:477–492.

Botha-Brink, J., and S. P. Modesto. 2011. A new skeleton of the therocephalian synapsid *Olivierosuchus parringtoni* from the Lower Triassic South African Karoo Basin. Palaeontology 54:591–606.

Crompton, A. W. 1955. A revision of the Scaloposauridae with special reference to kinetism in this family. Navorsinge van die Nasionale Museum 1:149–183.

Gauthier, J., A. G. Kluge, and T. Rowe. 1988. Amniote phylogeny and the importance of fossils. Cladistics 4:105–209.

Heever, J. van den. 1994. The cranial anatomy of the early Therocephalia (Amniota: Therapsida). Annals of the University of Stellenbosch 1:1–59.

Hopson, J. A., and H. Barghusen. 1986. An analysis of therapsid relationships: Pp. 83–106 *in* N. Hotton, P. D. MacLean, J. J. Roth, and E. C. Roth (eds.) The Ecology and Biology of Mammal-like Reptiles. Smithsonian Institution Press, Washington, D.C.

Hopson, J. A., and J. W. Kitching. 2001. A probainognathian cynodont from South Africa and the phylogeny of nonmammalian cynodonts. Bulletin of the Museum of Comparative Zoology 156:5–35.

Huttenlocker, A. K. 2013. The paleobiology of South African therocephalian therapsids (Amniota, Synapsida) and the effects of the end-Permian extinction on size, growth, and bone microstructure. Ph.D. Dissertation, University of Washington, Seattle, 414 pp.

Huttenlocker, A. K. 2014. Body size reductions in nonmammalian eutheriodont therapsids (Synapsida) during the end-Permian mass extinction. PLOS ONE 9:e87553.

Huttenlocker, A. K., and C. A. Sidor. 2016. The first karenitid (Therapsida, Therocephalia) from the upper Permian of Gondwana and the biogeography of Permo-Triassic therocephalians. Journal of Vertebrate Paleontology 36:e1111897. doi:10.1080/02724634.2016.1111897

Huttenlocker, A. K., C. A. Sidor, and K. D. Angielczyk. 2015. A new eutherocephalian from the upper Madumabisa Mudstone Formation (Upper Permian) of Zambia. Journal of Vertebrate Paleontology 35:e969400. doi:10.1080/02724634.2015.969400

Kemp, T. S. 1972a. The jaw articulation and musculature of the whaitsiid Therocephalia; pp. 213–230 in K. A. Joysey, and T. S. Kemp (eds.), Studies in Vertebrate Evolution. Oliver & Boyd, Edinburgh.

Kemp, T. S. 1972b. Whaitsiid Therocephalia and the origin of cynodonts. Philosophical Transactions of the Royal Society of London B 264:1–54.

Kemp, T. S. 2005. The Origin and Evolution of Mammals. Oxford University Press, Oxford: 331 pp.

Maier, W. 1999. On the evolutionary biology of early mammals with methodological remarks on the interaction between ontogenetic adaptation and phylogenetic transformation. Zoologischer Anzeiger 238:55–74.

Mendrez, C. H. 1974. A new specimen of *Promoschorhynchus platyrhinus* Brink 1954 (Moschorhinidae) from the *Daptocephalus*-zone (Upper Permian) of South Africa. Palaeontologia Africana 17:69–85.

Modesto, S., B. Rubidge, and J. Welman. 1999. The most basal anomodont therapsid and the primacy of Gondwana in the evolution of the anomodonts. Proceedings of the Royal Society of London. Series B: Biological Sciences 266:331–337.

Rubidge, B. S., and J. van den Heever. 1997. Morphology and systematic position of the dinocephalian *Styracocephalus platyrhynchus*. Lethaia, 30:157–168.

Rubidge, B. S., and J. W. Kitching. 2003. A new burnetiamorph (Therapsida: Biarmosuchia) from the lower Beaufort Group of South Africa. Palaeontology, 46:199–210.

Sidor, C. A. 2001. Simplification as a trend in synapsid cranial evolution. Evolution 55:1419–1442.

Sidor, C. A. 2003. Evolutionary trends and the origin of the mammalian lower jaw. Paleobiology 29: 605–640.

Sidor, C. A., and J. A. Hopson. 1998. Ghost lineages and "mammalness": assessing the temporal pattern of character acquisition in the Synapsida. Paleobiology 24:254–273.

Sidor, C. A., and J. Welman. 2003. A second specimen of *Lemurosaurus pricei* (Therapsida: Burnetiamorpha). Journal of Vertebrate Paleontology 23:631–642.

Sigurdsen, T., A. K. Huttenlocker, S. P. Modesto, T. B. Rowe, and R. Damiani. 2012. Reassessment of the morphology and paleobiology of the therocephalian *Tetracynodon darti* (Therapsida), and the phylogenetic relationships of Baurioidea. Journal of Vertebrate Paleontology 32:1113–1134.
